# miR-302d Competitively Binding with the lncRNA-341 Targets TLE4 in the Process of SSC Generation

**DOI:** 10.1155/2021/5546936

**Published:** 2021-06-08

**Authors:** Yani Zhang, Wenhui Zhang, Cai Hu, Yingjie Wang, Man Wang, Qisheng Zuo, Ahmed Kamel Elsayed, Yi Li, Bichun Li

**Affiliations:** ^1^College of Animal Science and Technology, Yangzhou University, Jiangsu Province Key Laboratory of Animal Breeding and Molecular Design, Yangzhou, 225009 Jiangsu, China; ^2^Joint International Research Laboratory of Agriculture and Agri-Product Safety of Ministry of Education of China, Yangzhou University, Yangzhou, 225009 Jiangsu, China; ^3^Faculty of Veterinary Medicine, Suez Canal University, 41522 Ismailia, Egypt; ^4^College of Computer Science and Technology, Wenzhou-Kean University, Wenzhou, 325035 Zhejiang, China

## Abstract

MicroRNAs (miRNAs) are essential factors in the reproductive process of poultry. Here, we found miR-302d is a potential differentiation and negative factor of chicken embryonic stem cells (ESCs) into spermatogonia stem cells (SSCs). The competition mechanism was carried out for the preliminary exploration to determine the relationship among miR-302d, lncRNA-341(interacting with miR-302d), and target gene TLE4. The results showed that lncRNA-341 can competitively bind to miR-302d to decrease the targeted binding of miR-302d and TLE4 which promotes the differentiation of chicken SSCs. Moreover, it is suggested that miR-302d may participate in the Wnt signaling pathway through TLE4.

## 1. Introduction

The reproductive performance of chicken production is affected by many factors, and genetic breeding is one of the most important factors. How to make use of genetic improvement to increase the production of chicken breeding has been the direction that many scholars have devoted to. At the same time, in the modern commercial production, artificial insemination (AI) has replaced natural and artificial auxiliary mating, greatly improving the production efficiency, to adapt to the development of modernization; thus, how to efficiently obtain the male germ cells of the cock has become one of the key techniques.

miRNA constitutes of a single endogenous small RNA with a length of about 22 nt that is widely found in humans, animals, plants, and viruses. Mature miRNA does not participate in protein coding, but it is an important gene regulatory factor involved in numerous fields. miRNA generally achieves posttranscriptional regulation by degrading target mRNA or blocking the translation of target mRNA affecting development, such as in cardiovascular formation [1], neural development [2], stem cell differentiation [3], apoptosis [4], and tumor formation [5].

Many reports about miRNA in mammalian germ cells are playing a crucial role in gonadal sex determination, male reproductive stem cell meiosis, spermatogonial cell differentiation, and other aspects. Fernández analyzed the miRNA expression patterns of embryonic male and female mouse primordial germ cells (PGCs), as well as gonadal cells [6], and found that the differences in the expression regulation of miR-199-214, miR-182-183-96, miR-34c-5p, and other key miRNA clusters all have a clear role in gonadal sex determination. Liu found that miR-34c may regulate the meiosis of male reproductive stem cells of milk goats and inhibit their proliferation [7]. miRNA can also coordinate with related signaling pathways to regulate the production of germ cells. Hiromitsu analyzed RNA-seq data to identify specific miRNA (miR-741-3p, miR-871-3p, and miR-880-3p) to mouse germ cells that are contiguous and adjacent to each other on the X chromosome [8] termed XmiRs (X care-linked miRNA). MiR-871 and miR-880 work together with WNT/beta-catenin signaling pathways to regulate the occurrence of testicular germ cells. Fu et al. found that miR-31-5p regulates the proliferation [9], DNA synthesis, and apoptosis of human SSCs through pak1-jazf1-cyclin A2 pathway.

However, the specific mechanism of miRNA function in birds is not fully understood in how it regulates germ cell development; thus, there are still many key miRNAs to be discovered and explored in poultry germ cells. Therefore, there are still a lot of gaps in the research on miRNA regulating the differentiation of avian male germ cells.

In the preliminary work, our lab has established the system for inducing the differentiation of ESCs into SSCs *in vitro* by RA (retinoic acid) and has been working on ways to improve the induction efficiency of this process. Therefore, we need to explore the important regulatory factors in this process, RNA-seq was performed in previous work, and the differential miR-302d were screened out. In this study, the function of miR-302d and its target gene were verified *in vivo* and *in vitro*, and the effect on Wnt signaling pathway has also been preliminarily explored. The ceRNA competition mechanism was also preliminarily explored to determine the relationship among miRNA, lncRNA, and target gene interactions. This study fills in the gap of miRNA in regulating the differentiation of avian male germ cells and improves reproductive modelling efficiency.

## 2. Materials and Methods

### 2.1. Bioinformatics Analysis

The ESCs at specific days (day 0, day 4, and day 10) induced by RA were collected, and RNA sequencing was performed. According to the RNA-seq data, differential miRNAs were screened (∣log2.Fold_change | >8 was the screening criteria). miR-302d, which plays an important role in germ cell differentiation, was identified as the research object. The target genes of gga-miR-302d (referred to as miR-302d) were analyzed and screened with online bioprediction software: http://www.targetscan.org/vert_71/(Targetscan); http://www.mirdb.org/(miRDB); we performed a Venn analysis of the screening results from these two databases and identified four common target genes. lncRNA transcripts were screened by exon number screening, length screening, known annotation screening, expression volume screening, and encoding potential screening of CNCI (Coding-Non-Coding Index), CPC (Coding Potential Calculator), PFAM (pfamscan), etc., lncRNA and miR-302d interaction analysis online bioprediction software: http://www.mirbase.org/(miRBase).

### 2.2. Vector Construction

miR-302d lentivirus expression vector and interference vector are provided by the Genomeditech Company (Shanghai, China). The expression vector was named miR-302d mimics, and the interference vector was named miRNA-inhibitor. Sequences were listed in supporting Table 1 and Table 2. The construction methods of TLE4 3′UTR wild-type and mutant pMIR-REPORT vector refer to the vector system protocol. pMIR-REPORT vector was purchased from Applied Biosystems (http://www.appliedbiosystems.com). Sequences were listed in supporting Table s8.

### 2.3. ESC Isolation and Induction Culture

ESCs were isolated from freshly fertilized eggs as previously described [10, 11]. ESCs were induced by medium containing 10^−5^ mol/L RA, and viruses were added with a titer of 5 × 10^8^ TU/mL on D0 induction. Cell morphology was observed every 2 days, and cells were collected from day 0, day 4, and day 10 for RNA, and cells were collected from day 4 and day 10 for immunofluorescence (IF) and flow cytometry (FCM). 30 eggs per treatment in 3 separate repeats of the experiment.

### 2.4. Embryo Injection Experiment

When the chicken embryos developed normally for 2.5 days, the blunt end was sterilized with 70% ethanol, and embryos were opened for microinjection into vessels. Each embryo was injected with 5 × 10^8^ TU/mL virus diluent of 2 *μ*L in total. After injection, 20 *μ*L of 1% penicillin and streptomycin were dropped into the injection site, sealed with medical tape, and incubated under normal conditions. The embryonic genital ridges were collected at 4.5 days, and testes separated at 18.5 days after incubation for RNA, Periodic Acid-Schiff stain (PAS), FCM, Western blot and other tests.

### 2.5. Luciferase Reporter Assay

DF-1 cells were cotransfected with pMIR-REPORT™ recombinant plasmid and pRL-TK at a ratio of 10 : 1. After 24 hours, remove the old medium, wash with PBS, add trypsin for digestion, and collec cells. Cells were suspended by 1× passive lysis buffer and moved into a 96-hole opaque/round bottom, enzyme-labeled plate. Luciferase assay substrate addition preceded reader analysis for firefly enzyme fluorescent value determination. STOPPING buffer was added accordingly for further fluorescent values of the kidney. For specific operations, refer to the instructions of the double luciferase reporter assay kit of Promega company. Finally, the relative fluorescence activity of the firefly value/renal value was calculated.

### 2.6. Quantitative Reverse Transcription PCR (qRT-PCR)

Total RNAs were extracted using Trizol, the FastQuant RT Kit (with gDNase), and the SuperReal PreMix Plus (SYBR Green) (TIANGEN Beijing, China). A 7500 qRT-PCR instrument system from Applied Biosystems (Carlsbad, California, USA) was used followed by Microsoft Excel software to analyze the data by 2^−ΔΔCt^ relative quantification method.

### 2.7. Immunofluorescence (IF)

The cells were fixed with 4% formaldehyde 10 min at room temperature (RT), washed 3 times with PBS for 5 mins each, 0.1% Triton X-100 for 10 min at RT, blocked for 60 min with 1% BSA at RT, incubated in primary antibody solution for overnight at 4°C, rabbit-CVH (dilution concentrations 1 : 400), mouse-ITG*β*1 (1 : 500) (all from Abcam, Cambridge, UK), washed 3 times with PBS for 5 mins each, incubated in secondary antibody solution for 2 hours at RT in the dark, goat anti-rabbit IgG (TRITC labeled, 1 : 1000), goat anti-mouse IgG (TRITC labeled, 1 : 2000) (all from Abcam, Cambridge, UK), washed 3 times with PBS, and stained with DAPI for 15 min. Images were captured with fluorescence microscope.

### 2.8. Flow Cytometry (FCM)

Cells were stained with antibodies against CVH, to assess the changes in PGC-related gene expression. To assess the level of SSC-related gene expression, cells were stained with ITG*β*1 (all from Abcam, Cambridge, UK). Samples were analyzed by BD FACS Aria flow cytometer (BD Biosciences, provided by the testing center of Yangzhou University).

### 2.9. PAS Staining

Embryos were fixed prior to performing gradient dehydration with different ethanol concentrations. The embryos were further transparentized with xylene, immersed in paraffin, and embedded with paraffin. Paraffin sections were dewaxed with xylene and rehydrated with different ethanol concentrations, then stained with PAS staining kit (Solarbio, Beijing, China) according to the manufacturer's instructions. More details can be found in our previous articles [10].

### 2.10. Statistical Analyses

Differences between groups were examined for statistical significance using Student's *t*-test or one-way ANOVA. *P* value < 0.05 was regarded as significant, and *P* value < 0.01 was regarded as extremely significant.

## 3. Results

### 3.1. Screening of *miRNA-302d* during RA-Induced ESC Differentiation into SSCs

**Figure 1:**
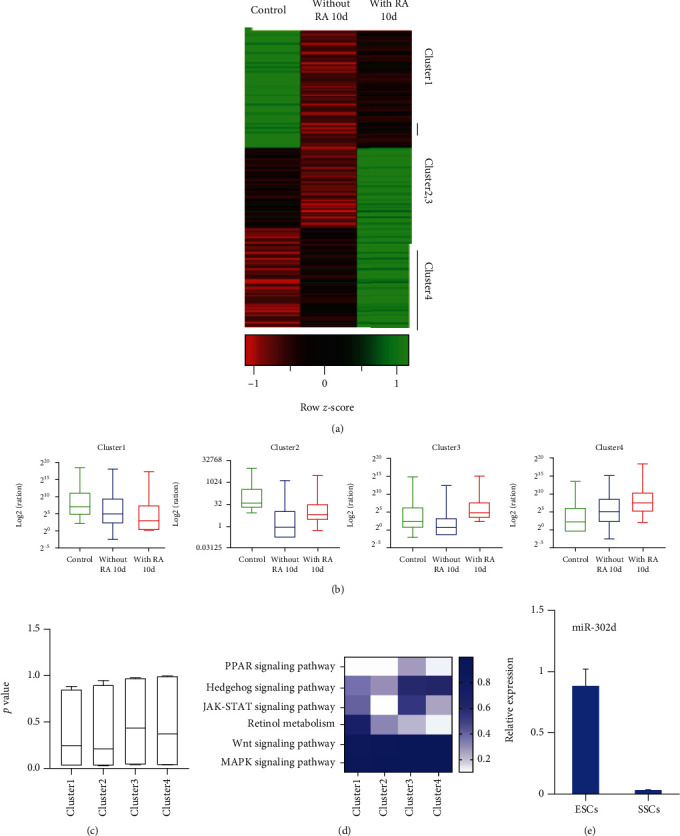
Screening of miRNA-302d during RA-induced ESC differentiation into SSCs. (a) Differential miRNA thermogram. (b) Box diagram analysis of *K*-means clustering. (c) GO analysis results. (d) KEGG analysis results. (e) Expression trends of miR-302d in ESCs and SSCs.

To explore the miRNA in RA-induced ESC differentiation into SSCs, ESCs, and SSCs, ESCs were treated with RA for 10 days. Cells were then processed for RNA sequencing. It was found that the miRNAs involving in the differentiation process were classified into four categories (Figures 1(a) and 1(b)). To identify specific groups involved in SSC differentiation, GO analysis was performed, and we found that the miRNA in the Cluster1 can be significantly enriched in the relevant GO items of germ cell development (Figure 1(c)). Comparing with other groups, miRNA in the Cluster1 mainly involved in the formation of SSCs. To further clarify the functions of miRNA in various groups, we conducted KEGG pathway enrichment analysis. We found that the miRNA in the Cluster1 is significantly enriched in the SSC formation of related signaling pathways, such as Wnt signaling pathway [12], JAK-STAT signaling pathway [13], and MAPK signaling pathway [14] (Figure 1(d)), showing that among the four miRNA clusters, miRNAs in the Cluster1 were more likely to participate in the SSC formation process. Meanwhile, we found these signaling pathways are regulated by miRNA302d target genes. And qRT-PCR showed that the expression of miRNA302d in ESCs was higher than that of SSCs (Figure 1(e)), suggesting that miRNA302d may play an important role in SSC formation.

### 3.2. miR-302d Inhibits the Differentiation of Chicken ESCs into SSCs

**Figure 2:**
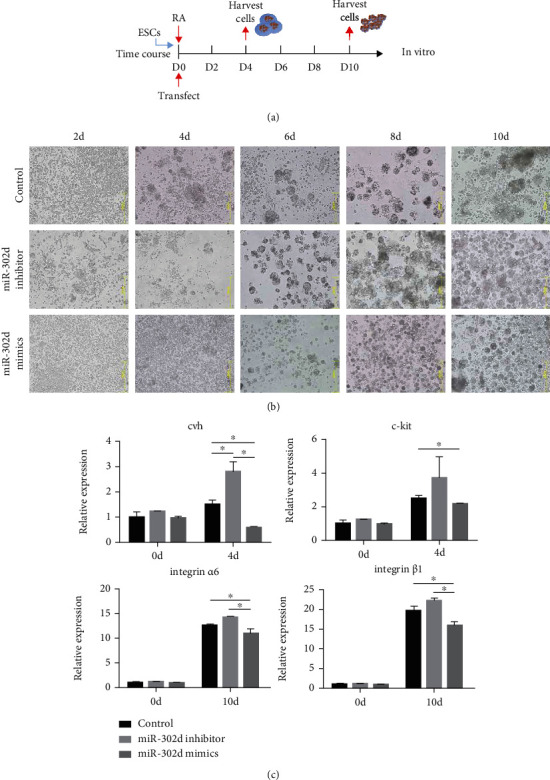
miR-302d function verification in vitro. (a) Experimental scheme in vitro. (b) Cell morphology every two days, control: inducing by RA; miR-302d inhibitor: based on RA induction, miR-302d inhibitor lentivirus was infected on day 0; miR-302d mimics: based on RA induction, miR-302d mimics lentivirus was infected on day 0. (c) mRNA expression levels of marker genes were detected on day 4 and day 10; cvh and c-kit were used as PGC maker genes in chicken, integrin *α*6 and integrin *β*1 were SSC maker genes, and *β*-actin was the reference gene.

**Figure 3:**
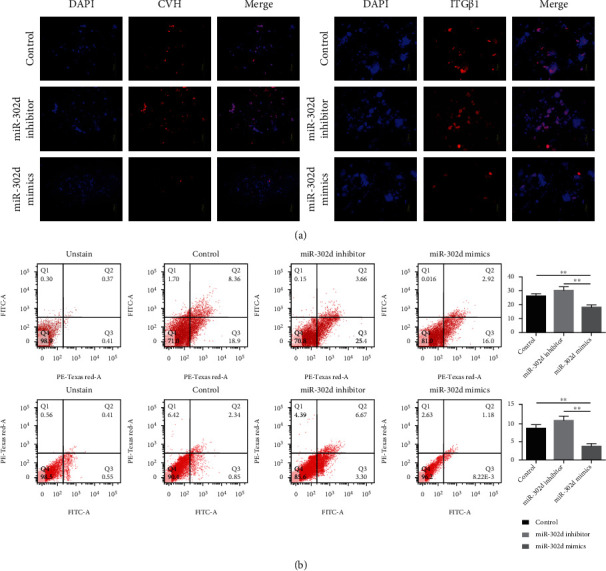
miR-302d function verification in vitro. (a) Immunofluorescence test on day 4 and day 10. (b) Flow cytometry analysis on day 4 and day 10, and quantification of positive cells; here, PE-Texas Red means TRITC channel.

To further determine the role of miRNA-302d in regulating ESC differentiation into SSCs, we also used RA induction system which could induce ESC differentiation into SSCs. We observed cell morphology every 2 days, and marker genes were detected during embryonic bodies (EBs) and SSC-like cell formation (Figure 2(a)). Since the EBs appear on day 4 and the SSC-like cell appears on day 10 *in vitro*, we choose these two timepoints. Briefly describe: we treated ESCs with miRNA-302d inhibitor or miRNA-302d mimics and measure maker gene expression. EBs in the inhibitor group were significantly more than other groups, but the mimics group showed the opposite results (Figure 2(b)). The expression of reproductive marker genes was significantly increased in the cells treated by miRNA-302d inhibitor and decreased in the cells treated with miRNA-302d mimics (Figure 2(c), Table s1). In IF, the expression of marker proteins in the mimics group decreased, while in the inhibitor group, it was increased (Figure 3(a)). Flow cytometry analysis (FCM) showed that the positive cell rate in the mimics group was significantly lower than that in the control group, while the inhibitor group had no significant difference compared with the control group but had an upward trend (Figure 3(b)). The above results indicated that miR-302d inhibits the differentiation of chicken ESCs into SSCs *in vitro.*

**Figure 4:**
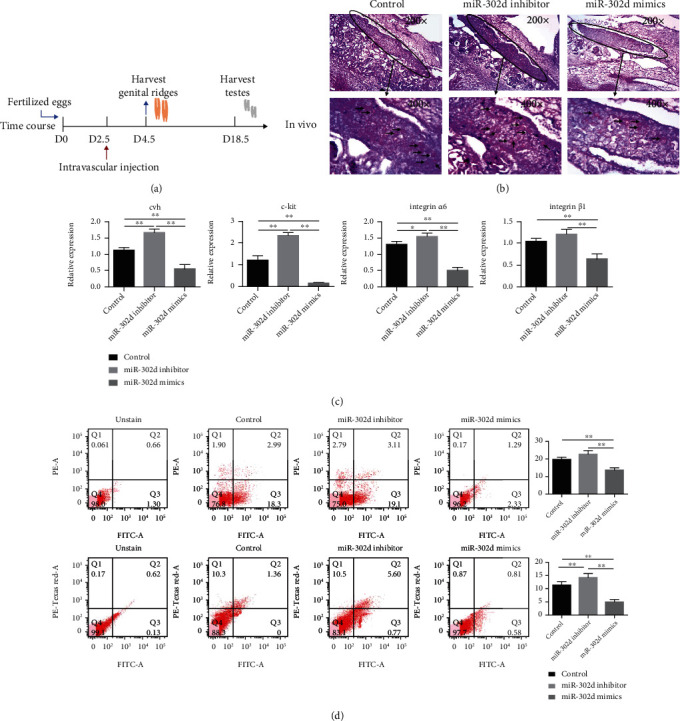
miR-302d function verification in vivo. (a) Experimental scheme in vivo. (b) PAS staining of day 4.5 embryos, in the oval is the genital ridge; ↑ the arrows point to PGCs. (c) Marker genes were detected on day 4.5 genital ridge and day 18.5 testes. (d) Flow cytometry analysis on day 4.5 genital ridge and day 18.5 testes and quantification of positive cells.

We then injected miRNA-302d inhibitor or miRNA-302d mimics viruses into 2.5-day-old chicken embryo vessels (Figure 4(a)). 4.5-day-old chicken embryos were harvested for PAS. Genital ridges were observed and developed normally. The number of PGCs increased in the inhibitor group and decreased in the mimics group compared with the control group (Figure 4(b)). We harvested 4.5-day-old genital ridges and 18.5-day-old testes and examined the mRNA expression of reproduction-related genes *cvh*, *c-kit*, *integrin α6*, and *integrinβ1*. The miRNA-302d mimics significantly inhibited the expression of these marker genes (Figure 4(c), Table s2). Likewise, FCM showed that the number of marker-protein-positive cells in the mimics group was significantly lower than that in the control group (Figure 4(d)). The above results indicated that miR-302d had an inhibitory effect on SSC differentiation in chicken.

### 3.3. miR-302d Can Bind to the 3′UTR of Target Gene TLE4 and Regulate Its Expression

**Figure 5:**
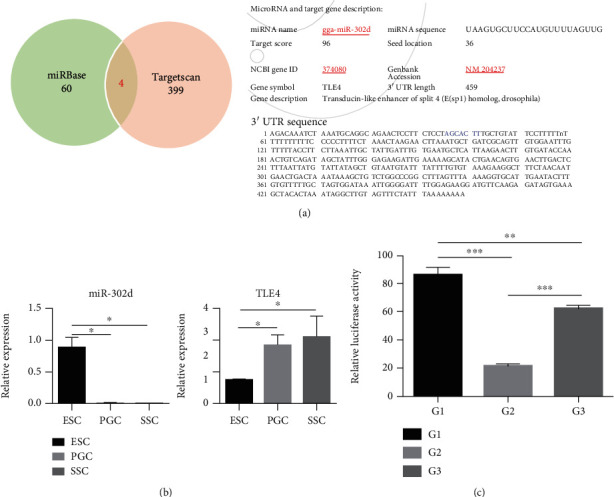
Screening and verification of target gene TLE4. (a) Venn diagram of biometric prediction software analysis result; detail information and 3′UTR sequence of TLE4. (b) Expression level of miR-302d and TLE4 in ESCs, PGCs, and SSCs. (c) Dual-luciferase reporting assay; G1: pmiR-tle4-wt group (use TLE4 3′UTR wild-type pmiR-REPORT vector to transfect DF-1 cells), G2: pmiR-tle4-wt+miR-302d mimics group (use TLE4 3′UTR wild-type pmiR-REPORT vector and miR-302d mimics to transfect DF-1 cells), G3: pmiR-tle4-mt+miR-302d mimics group (use TLE4 3′UTR mutant-type pmiR-REPORT vector and miR-302d mimics to transfect DF-1 cells, TLE4 3′UTR mutant-type vector lacks binding site: AGCACTT).

According to gga-miR-302d (MIMAT0003360) sequence, UAAGUGCUUCCAUGUUUUAGUU, we used multiple online biological software to analyze target genes and ranked them by scores (Figure 5(a)). Among these, four common target genes were identified: *MYT1L*, *ELAVL2*, *TLE4*, and *HLF* (Table s3). *TLE4*, which has the highest target score of 96, is a target gene that maintains stem cell pluripotency. We speculated that it may play an important role in the process of chicken SSC differentiation. Therefore, we carried out targeted verification on *TLE4*.

According to the expression profile results (Figure 5(b)), miR-302d and *TLE4* showed opposite expression trends in ESCs, PGCs, and SSCs. miR-302d had the highest expression in ESCs, but TLE4 expressed more in SSCs than ESCs and PGCs. In the dual-luciferase reporting assay (Figure 5(c)). The results indicated that miR-302d inhibited the expression of TLE4 mRNA, but when the binding site of miR-302d and *TLE4* was deleted, reducing the binding of miR-302d and *TLE4* and making up for the inhibition of *TLE4* mRNA expression. In conclusion, TLE4 is the target gene of miR-302d, and miR-302d suppresses its expression.

### 3.4. TLE4 Promotes the Differentiation of Chicken SSCs

**Figure 6:**
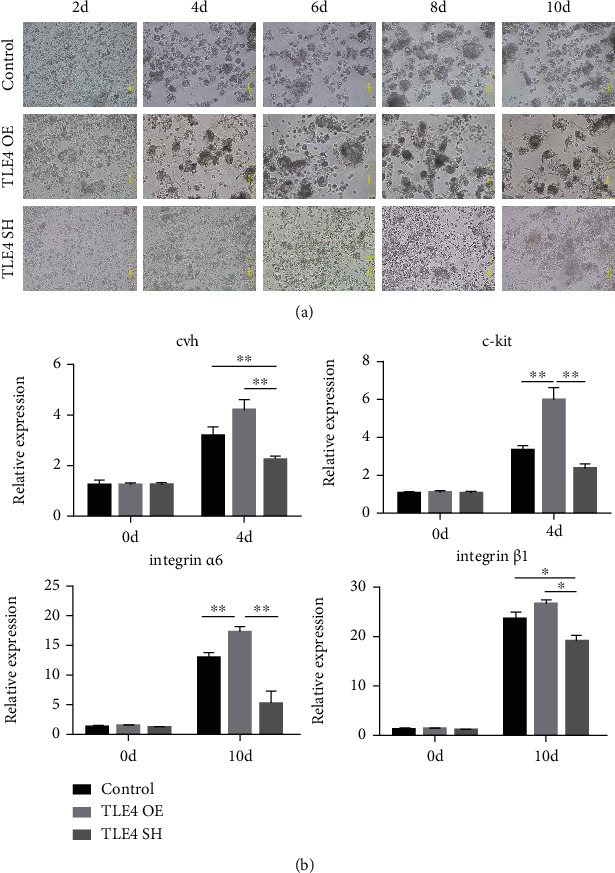
TLE4 function verification in vitro. (a) Cell morphology every two days; TLE4 OE: TLE4 overexpression; TLE4 SH: TLE4 suppression. (b) Marker genes were detected on day 4 and day 10.

**Figure 7:**
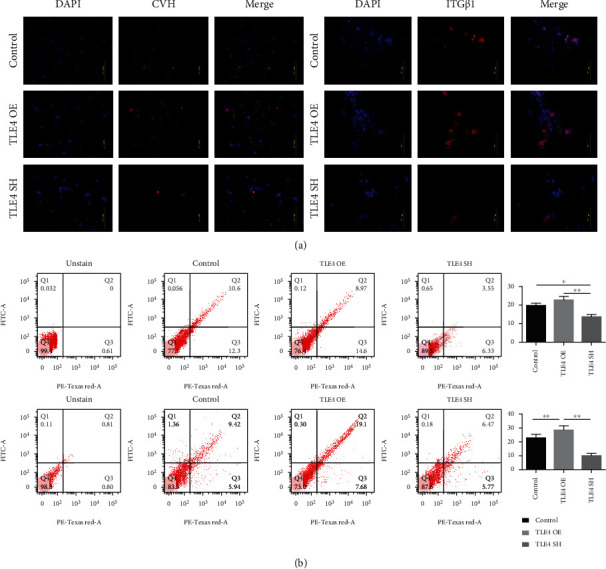
TLE4 function verification in vitro. (a) Immunofluorescence test on day 4 and day 10. (b) Flow cytometry analysis on day 4 and day 10, and quantification of positive cells; here, PE-Texas Red means TRITC channel.

We examined the function of *TLE4* in vitro and *in vitro* and *in vivo*. We treated ESCs with virus for overexpression and inhibition. We then observed cell morphology every two days, and marker genes were detected. EBs in overexpression (OE) group were significantly more than that of other groups (Figure 6(a)). The qRT-PCR results also showed that the expression of marker genes in the OE group was significantly higher than that in other groups, while the SH group was significantly downregulated (Figure 6(b), Table s4). In IF, the expression of marker proteins in SH group was decreased, while that in the OE group was increased (Figure 7(a)). FCM showed that the amount of CVh^+^ and integrin^+^ cells in the SH group was significantly lower than that in the control group and OE group, while the OE group was significantly higher than that in the control group on day 10 (Figure 7(b)). The above results indicated that *TLE4* promotes the differentiation of chicken ESCs into SSCs in vitro.

**Figure 8:**
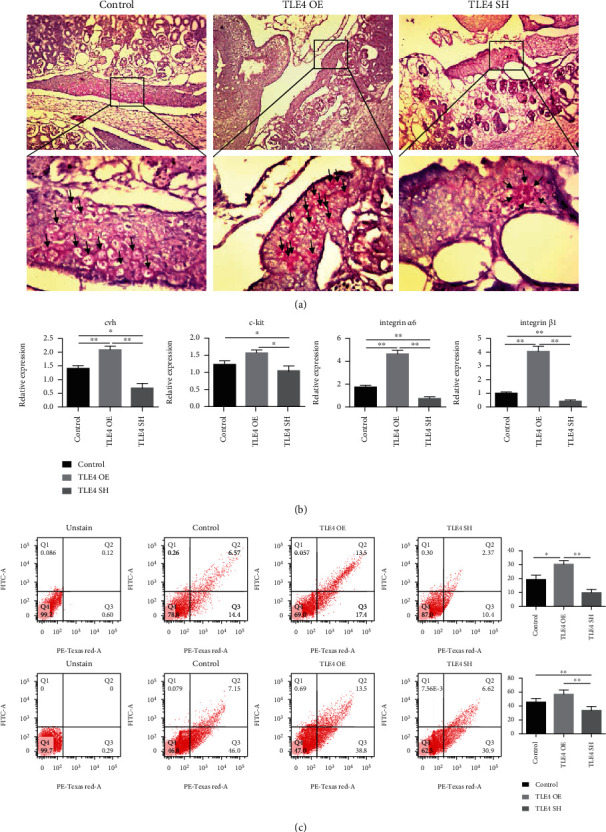
TLE4 function verification in vivo. (a) PAS staining of day 4.5 embryos, in the oval is the genital ridge; ↑ the arrows point to PGCs. (b) Marker genes were detected on day 4.5 genital ridge/day 18.5 testes. (c) Flow cytometry analysis on day 4.5 genital ridge/day 18.5 testes and quantification of positive cells.

We harvested 4.5-day-old chicken embryos for PAS staining. At high magnification, the number of PGCs increased in the OE group and decreased in the SH group compared with the control group (Figure 8(a)). We detected the mRNA expression of reproduction-related genes, and the results showed that the SH group significantly inhibited the expression of marker genes, while the OE group promoted the expression (Figure 8(b)), and the qRT-PCR data is showed in Table s5. Likewise, FCM showed that the rate of marker-protein-positive cells in the SH group was significantly lower than that in the OE group (Figure 8(c)). The above results indicated that *TLE4* had a positive effect on SSC differentiation in chicken.

### 3.5. miR-302d Can Affect the Expression of Node Genes Directly or Indirectly Interacting with TLE4 on the Wnt Signaling Pathway

It has been shown that *TLE4* plays an important role in the signaling pathway related to germ cell differentiation [15]. Proteins expressed in certain nodes of the Wnt pathway have been shown in mice to bind directly or indirectly to the TLE4 protein [15–17]. To explore whether miR-302d can target TLE4 and affect the expression of node genes in the signaling pathway, related node genes in Wnt signaling were selected for analysis, including *Tcf*, *Lef*, *β-catenin*, *Axin1*, and *Apc*.

**Figure 9:**
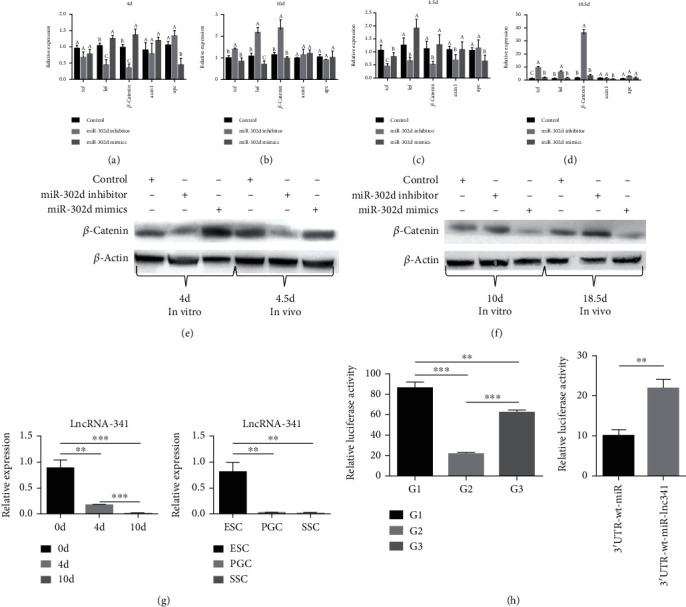
Wnt signaling pathway verification and ceRNA verification. (a) qRT-PCR result of related genes in Wnt signaling pathway of day 4 in vitro. (b) qRT-PCR result of day 10 in vitro. (c) qRT-PCR result of day 4.5 in vivo. (d) qRT-PCR result of day 18.5 in vivo. (e) Western blot test in vitro. (f) Western blot test in vivo. (g) lncRNA-341 expression test. (h) Dual-luciferase reporting assay; G1: pmiR-tle4-wt wild type; G2: pmiR-tle4-wt+miR-302d mimics; G3:pmiR-tle4-mt+miR-302d mimics; 3′UTR-wt-miR: pmiR-report-wt+pRL-TK+miRNA-302d mimics; 3′UTR-wt-miR-lnc341: pmiR-report-wt+pRL-TK+miRNA-302d mimics+pcDNA3.1-lnc341.

4-day and 10-day cells were harvested and analyzed for mRNA and protein expression involving miR-302d functional verification in vitro. On day 4, the expression of *Lef* and *β-catenin* was significantly upregulated in miR-302d mimics group compared with the control group, *Apc* was significantly downregulated in mimics group compared with the control group, and *Tcf* and *Axin1* had no significant difference (Figure 9(a); Table s6). Western blot showed that the expression of *β*-catenin in the mimics group was more than the control group (Figure 9(e)). On day 10, *Tcf*, *Lef*, and *β-catenin* were significantly upregulated in the inhibitor group compared with the control group, but *Axin1* and *Apc* had no significant difference (Figure 9(b); Table s6). Western blot test showed that the expression of *β*-catenin in the mimics group to be far less than the control (Figure 9(f)).

In vivo, embryonic genital ridges were harvested for qRT-PCR test on day 4.5, *Tcf*, *Lef*, *β-catenin*, and *Axin1* were significantly downregulated in the inhibitor group compared with the control group, but *Apc* had no significant difference (Figure 9(c); Table s7). Western blot analysis showed that *β*-catenin expression in the inhibitor group was less than the control group (Figure 9(e)). Testes were harvested for qRT-PCR test on day 18.5; *Tcf*, *Lef*, and *β-catenin* were significantly upregulated in the inhibitor group compared with the control group, but *Axin1* and *Apc* had no significant difference (Figure 9(d); Table s7). Western blot analysis showed the expression of *β*-catenin in the mimics group was less than the control (Figure 9(f)). As a result, miR-302d can affect the expression of node genes directly or indirectly interacting with TLE4 on the Wnt signaling pathway, the overexpression of miR-302d made the expression of *Lef*, *β-catenin*, *Axin1*, and *Tcf* fluctuate during the differentiation of ESCs into SSCs.

### 3.6. lncRNA-341 Competes with miR-302d to Bind the Target Gene TLE4

After further RNA-seq database analysis, it was found that a total of 269 lncRNAs were correlated with the downregulation of miR-302d expression (Figure S1A). We predicted and individually analyzed these 269 lncRNAs and found that two lncRNAs were interacting with miR-302d: lncRNA-341 and lncRNA-1784 (Figure S1B). We verified these two lncRNAs and found no difference in the expression trend of lncRNA-1784 between ESCs, PGCs, and SSCs. The downregulation trend of lncRNA-341 expression between ESCs, PGCs, and SSCs was consistent with the sequencing results, indicating that the regulation mechanism of lncRNA-1784 expression may not be consistent in experiments in vivo and in vitro. Therefore, we chose lncRNA-341 for further investigation.

lncRNA -341 was significantly downregulated between ESCs, PGCs, and SSCs, which was consistent with the trends by time in vitro (Figure 9(g)). To further elucidate if lncRNA-341 could affect the targeted regulation of TLE4 by miR-302d, we conducted an lncRNA-341 expression vector cotransfected, respectively, with miR-302d mimics and TLE4 double-luciferase reporter vector systems. In double luciferase report analysis, two groups were set up. The 3′UTR -wt-miR-lnc341 group was significantly upregulated compared to the 3′UTR-wt-miR group (Figure 9(h)). Combined with the previous miR-302d binding of target gene 3′UTR experiment analysis, it showed that lncRNA-341 could bind miR-302d and block its targeted binding to TLE4, thus upregulating TLE4 expression.

## 4. Discussion

Over the past few decades, chicken has been widely used as a model animal in both commercial breeding and scientific research, contributing to the development of the global poultry industry and providing a wealth of information for basic research. Improving the quality and productivity of male germ cells could bring huge benefits to commercial production and could be widely applied to other species, including humans. Although many studies have shown that RA can induce different stem cell donors to differentiate into SSCs in vitro [18, 19], the efficiency is not stable due to differences in the culture and induction system. The regulation mechanism of male germ cell production is complex, rendering it a challenge to harvest induced male germ cells with high purity and quality continuously in vitro, which makes it difficult to be applied in actual production. In our previous studies, we found many key genes, which may be regulated by some noncoding RNAs, such as lncRNA and miRNA. Therefore, it is necessary to explore these factors, to delineate the mechanisms and potentially improve induction efficiency.

**Figure 10:**
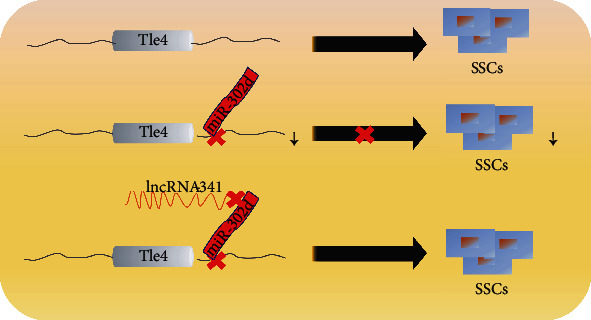
Interactive pattern of miR-302d and lncRNA-341 and Tle4.

As is known, KIT receptor expression is activated during spermiogenesis. In mice undifferentiated spermatagonial cells, the functional damage of miR-221/222 gathered on the X chromosome would convert the KIT^−^ to KIT^+^ and cause the loss of sperm regeneration ability in stem cells [20]. In spermatogonium, KIT mRNA and protein abundances are influenced by miR-221/222, though RA can decrease miR-221/222 abundance to induce undifferentiated SSCs to KIT^+^. However, overexpression of miR-221/222 inhibited the RA induction process, resulting in the inability of spermatogonial cells to differentiate in vivo, indicating that miR-221/222 played a crucial role in maintaining the undifferentiated status of mammalian spermatogonial cells by inhibiting the expression of KIT. Aside from germ cell differentiation, most miR-302 family members are related to mammalian breast cancer [21], reproductive cell carcinoma [22], DNA damage [23], and fibroblast differentiation [24]. These studies have shown that miR-302 family has high specificity or differentiation expression patterns in mammalian ESCs, and it is consistent with our RNA-seq data in chicken which shows the differentially high expression of miR-302d in chicken ESCs. A small number of studies have also shown that gga-miR-181-5p, gga-miR-2127, and gga-miR-302/367 clusters play a leading role in the regulation of proliferation of avian primitive germ cells [25]. Both gga-miR-302b and gga-miR-17-5p can regulate glucose phosphate isomerase and affect the proliferation of PGCs [26] which suggests that miR-302d may play an important role in the differentiation of chicken male germ cells. In this study, our result proved this hypothesis, miR-302d can inhibit differentiation of ESCs into SSCs both in vivo and in vitro, and lncRNA-341 can interact with miR-302d to reduce the targeted binding between miR-302d and TLE4 (Figure 10), and the targeting regulation of TLE4 by miR-302d can affect the expression of key genes in Notch and Wnt signaling pathways.

However, due to the complex nature of the miR-302d regulation network, although we have preliminarily verified the relationship of lncRNA-341-miR302-TLE4, whether it is affected by other factors requires further exploration. For example, RNA pull-down can be used to detect whether other protein regulators are involved. Further exploration must be undertaken to elucidate the regulatory network. Overall, miRNA302d plays an important role in the differentiation of chicken male germ cells and is a significant regulatory factor.

## Figures and Tables

**Table 1:** pri-miR-302d primer sequences.

Primer	Primer sequence (5′-3′)	Size/bp
F	GAACCGGTGCGGCCGCCTGGATGTTGGAACAGAAGAAC	38 bp
R	CGATCGCAGATCCTTGTAGCCGAGAAGGATGAAAACC	37 bp

**Table 2:** shRNA oligo primer sequences for miR-302d.

Primer	Primer sequence (5′-3′)	Size/bp
F	GATCCGACGGCGCTAGGATCATCAACCAACTAAAACATGATCTGAAGCACTTACAAGTATTCTGGTCACAGAATACAACCAACTAAAACATGATCTGAAGCACTTACAAGATGATCCTAGCGCCGTCTTTTTTG	134 bp
R	AATTCAAAAAAGACGGCGCTAGGATCATCTTGTAAGTGCTTCAGATCATGTTTTAGTTGGTTGTATTCTGTGACCAGAATACTTGTAAGTGCTTCAGATCATGTTTTAGTTGGTTGATGATCCTAGCGCCGTCG	134 bp

## Data Availability

The (data type) data used to support the findings of this study are included within the article and the supplementary information file(s).

## Supplementary Materials

Supplementary Materials Table s1: qRT-PCR data of miR-302d function verification in vitro. Table s2: qRT-PCR data of miR-302d function verification in vivo. Table s3: scores of candidate genes. Table s4: qRT-PCR data of TLE4 function verification in vitro. Table s5: qRT-PCR data of TLE4 function verification in vivo. Table s6: qRT-PCR data of Wnt signaling pathway verification in vitro. Table s7: qRT-PCR data of Wnt signaling pathway verification in vivo. Table s8: the sequence of TLE4 3′UTR. Figure S1: lncRNA screening and analyses. A, 269 lncRNAs correlated with the downregulation of miR-302d expression. B, information of two lncRNAs interacted with miR-302d, lncRNA-341, and lncRNA-1784.
