# Supplementary material for: miR-302d Competitively Binding with the lncRNA-341 Targets TLE4 in the Process of SSC Generation
Source: Stem Cells Int. 2021 Jun 8;2021:5546936. doi: 10.1155/2021/5546936 (PMC8205581; doi:10.1155/2021/5546936)
Supplement: Supplementary Materials — Table s1: qRT-PCR data of miR-302d function verification in vitro. Table s2: qRT-PCR data of miR-302d function verification in vivo. Table s3: scores of candidate genes. Table s4: qRT-PCR data of TLE4 function verification in vitro. Table s5: qRT-PCR data of TLE4 function verification in vivo. Table s6: qRT-PCR data of Wnt signaling pathway verification in vitro. Table s7: qRT-PCR data of Wnt signaling pathway verification in vivo. Table s8: the sequence of TLE4 3′UTR. Figure S1: lncRNA screening and analyses. A, 269 lncRNAs correlated with the downregulation of miR-302d expression. B, information of two lncRNAs interacted with miR-302d, lncRNA-341, and lncRNA-1784. [file 5546936.f1.docx]

Table s1 qRT-PCR data of miR-302d function verification in vitro

| Gene | Day | Group | Relative expression level |
| --- | --- | --- | --- |
| cvh | 4 | control | 1.51±0.17 |
|  |  | miR-302d inhibitor | 2.81±0.38 |
|  |  | miR-302d mimics | 0.60±0.04 |
| c-kit | 4 | control | 2.51±0.17 |
|  |  | miR-302d inhibitor | 3.74±1.24 |
|  |  | miR-302d mimics | 2.18±0.04 |
| integrin α6 | 10 | control | 12.66±0.17 |
|  |  | miR-302d inhibitor | 14.28±0.19 |
|  |  | miR-302d mimics | 10.98±0.89 |
| integrin β1 | 10 | control | 19.66±1.17 |
|  |  | miR-302d inhibitor | 22.28±0.59 |
|  |  | miR-302d mimics | 15.98±0.89 |

Table s2qRT-PCR data of miR-302d function verification in vivo

| Gene | Day | Group | Relative expression level |
| --- | --- | --- | --- |
| cvh | 4.5 | control | 1.12±0.09^c^ |
|  |  | miR-302d inhibitor | 1.67±0.11 |
|  |  | miR-302d mimics | 0.55±0.14 |
| c-kit | 4.5 | control | 1.22±0.19 |
|  |  | miR-302d inhibitor | 2.37±0.11 |
|  |  | miR-302d mimics | 0.15±0.04 |
| integrin α6 | 18.5 | control | 1.31±0.08 |
|  |  | miR-302d inhibitor | 1.56±0.07 |
|  |  | miR-302d mimics | 0.50±0.09 |
| integrin β1 | 18.5 | control | 1.05±0.06 |
|  |  | miR-302d inhibitor | 1.21±0.11 |
|  |  | miR-302d mimics | 0.65±0.11 |

Table s3scores of candidate genes

| Gene | Score | Reference |
| --- | --- | --- |
| TLE4 | 96 | maintains stem cell pluripotency |
| MYT1L | 93 | belongs to the neuron cell gene |
| ELAVL2 | 82 | mainly associate with gastric cancer liver metastasis |
| HLF | 80 | mammary gland epithelial gene |

Table s4 qRT-PCR data ofTLE4 function verification in vitro

| Gene | Day | Group | Relative expression level |
| --- | --- | --- | --- |
| cvh | 4 | control | 3.45±0.35 |
|  |  | TLE4 OE | 4.38±0.43 |
|  |  | TLE4 SH | 2.39±0.12 |
| c-kit | 4 | control | 3.50±0.39 |
|  |  | TLE4 OE | 6.01±0.81 |
|  |  | TLE4 SH | 2.48±0.20 |
| integrin α6 | 10 | control | 13.45±1.12 |
|  |  | TLE4 OE | 18.59±1.09 |
|  |  | TLE4 SH | 5.19±2.69 |
| integrin β1 | 10 | control | 18.11±2.69 |
|  |  | TLE4 OE | 25.77±2.15 |
|  |  | TLE4 SH | 12.19±3.48 |

Table s5 qRT-PCR data ofTLE4 function verification in vivo

| Gene | Day | Group | Relative expression level |
| --- | --- | --- | --- |
| cvh | 4.5 | control | 1.24±0.16 |
|  |  | TLE4 OE | 1.96±0.12 |
|  |  | TLE4 SH | 0.75±0.07 |
| c-kit | 4.5 | control | 1.12±0.09 |
|  |  | TLE4 OE | 1.33±0.12 |
|  |  | TLE4 SH | 0.75±0.21 |
| integrin α6 | 18.5 | control | 1.05±0.11 |
|  |  | TLE4 OE | 4.36±0.56 |
|  |  | TLE4 SH | 0.55±0.09 |
| integrin β1 | 18.5 | control | 1.11±0.10 |
|  |  | TLE4 OE | 3.68±0.22 |
|  |  | TLE4 SH | 0.26±0.22 |

Table s6 qRT-PCR data of Wnt signaling pathwayverification in vitro.

| Gene | Day | Group | Relative expression level |
| --- | --- | --- | --- |
| Tcf | 4 | control | 0.95±0.10 |
|  |  | miR-302d inhibitor | 0.68±0.17 |
|  |  | miR-302d mimics | 0.78±0.14 |
|  |  | control | 1.02±0.08 |
|  | 10 | miR-302d inhibitor | 1.40±0.06 |
|  |  | miR-302d mimics | 0.85±0.17 |
| Lef | 4 | control | 1.02±0.09 |
|  |  | miR-302d inhibitor | 0.45±0.15 |
|  |  | miR-302d mimics | 1.26±0.08 |
|  |  | control | 1.08±0.13 |
|  | 10 | miR-302d inhibitor | 2.17±0.0.22 |
|  |  | miR-302d mimics | 0.72±0.14 |
| β-catenin | 4 | control | 0.98±0.09 |
|  |  | miR-302d inhibitor | 0.36±0.12 |
|  |  | miR-302d mimics | 1.37±0.18 |
|  |  | control | 1.15±0.09 |
|  | 10 | miR-302d inhibitor | 2.39±0.36 |
|  |  | miR-302d mimics | 0.98±0.08 |
| Axin1 | 4 | control | 0.90±0.23 |
|  |  | miR-302d inhibitor | 0.79±0.31 |
|  |  | miR-302d mimics | 1.19±0.11 |
|  |  | control | 0.99±0.06 |
|  | 10 | miR-302d inhibitor | 1.15±0.25 |
|  |  | miR-302d mimics | 1.21±0.16 |
|  |  | control | 1.05±0.12 |
| Apc | 4 | miR-302d inhibitor | 1.33±0.17 |
|  |  | miR-302d mimics | 0.44±0.21 |
|  |  | control | 1.03±0.13 |
|  | 10 | miR-302d inhibitor | 0.91±0.04 |
|  |  | miR-302d mimics | 1.03±0.29 |

Table s7 qRT-PCR data of Wnt signaling pathwayverification in vivo.

| Gene | Day | Group | Relative expression level |
| --- | --- | --- | --- |
| Tcf | 4.5 | control | 1.05±0.21 |
|  |  | miR-302d inhibitor | 0.46±0.09 |
|  |  | miR-302d mimics | 0.82±0.13 |
|  |  | control | 1.09±0.13 |
|  | 18.5 | miR-302d inhibitor | 9.24±1.15 |
|  |  | miR-302d mimics | 1.56±0.20 |
| Lef | 4.5 | control | 1.25±0.27 |
|  |  | miR-302d inhibitor | 0.65±0.12 |
|  |  | miR-302d mimics | 1.91±0.33 |
|  |  | control | 1.01±0.23 |
|  | 18.5 | miR-302d inhibitor | 5.86±1.12 |
|  |  | miR-302d mimics | 1.51±0.33 |
| β-catenin | 4.5 | control | 1.11±0.29 |
|  |  | miR-302d inhibitor | 0.52±0.12 |
|  |  | miR-302d mimics | 1.27±0.38 |
|  |  | control | 1.25±0.21 |
|  | 18.5 | miR-302d inhibitor | 36.1±2.06 |
|  |  | miR-302d mimics | 3.10±1.14 |
| Axin1 | 4.5 | control | 1.09±0.13 |
|  |  | miR-302d inhibitor | 0.69±0.21 |
|  |  | miR-302d mimics | 1.09±0.29 |
|  |  | control | 1.17±0.26 |
|  | 18.5 | miR-302d inhibitor | 1.25±0.25 |
|  |  | miR-302d mimics | 0.45±0.16 |
|  |  | control | 1.05±0.11 |
| Apc | 4.5 | miR-302d inhibitor | 1.15±0.31 |
|  |  | miR-302d mimics | 0.64±0.23 |
|  |  | control | 1.04±0.23 |
|  | 18.5 | miR-302d inhibitor | 2.20±0.91 |
|  |  | miR-302d mimics | 1.54±0.49 |

Table s8The sequence of TLE4 3’UTR

| TLE4 3’UTRsequence(5’-3’) |  |
| --- | --- |
| AGACAAATCTAAATGCAGGCAGAACTCCTTCTCCTAGCACTTTGCTGTATTCCTTTTTNTTTTTTTTTTCCCCCTTTTCTAAACTAAGAACTTAAATGCTGATCGCAGTTGTGGAATTTGTTTTTACCTTCTTAAATTGCTATTGATTTGTGAATGCTCATTAAGAACTTGTGATACCAAACTGTCAGATAGCTATTTGGGAGAAGATTGAAAAAGCATACTGAACAGTGAACTTGACTCTTTAATTATGTATTATAGCTGTAATGTATTTATTTTGTGTAAAGAAGGCTTTCTAACAATGAACTGACTAAATAAAGCTGTCTGGCCCGGCTTTAGTTTAAAAGGTGCATTGAATACTTTGTGTTTTTGCTAGTGGATAAATTGGGGATTTTGGAGAAGGATGTTCAAGAGATAGTGAAAGCTACACTAAATAGGCTTGTAGTTTCTATTTAAAAAAAA | The predicted binding site is shown in yellow |


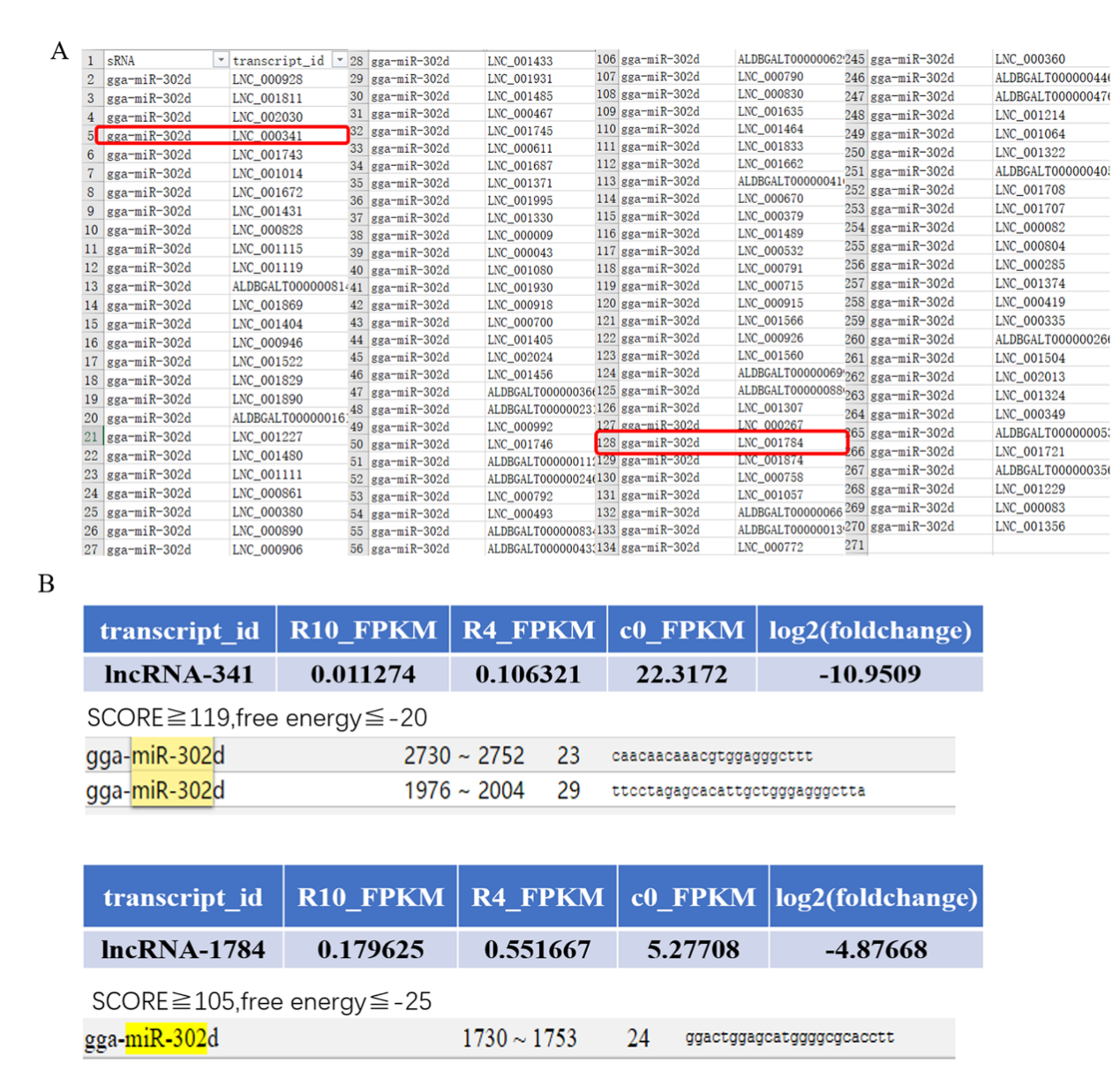


**Fig. S1 lncRNA screening and analyze.** A, 269 lncRNAscorrelated with the down-regulation of miR-302d expression. B, information of two lncRNAs interacted with miR-302d, lncRNA-341 and lncRNA -1784.
